# Supplementary material for: Correlation between maternal and umbilical cord 25-hydroxy-vitamin D levels over a range of values. A prospective observational study from the United Arab Emirates
Source: PLoS One. 2024 Apr 23;19(4):e0299862. doi: 10.1371/journal.pone.0299862 (PMC11037530; doi:10.1371/journal.pone.0299862)
Supplement: Attachment — Submitted filename: Response to reviewers, PLOS one.pdf [file pone.0299862.s001.pdf]

Cover letter II, Answers to reviewers.

Dear Editor and Reviewers,
Thank you for your precious, careful, and constructive feedback. We excuse the delay in our corrections. Per your suggestions, we have reviewed the manuscript; please find the changes in red.

Regarding the statistical reviewing we have tried to answer your questions and suggestions to the best of our ability together with our statistician and hope we have addressed your queries to satisfaction. Although no reviewer stated that the statistical analysis was not appropriately and rigorously conducted, we nevertheless made calculations of our data non-parametric but the results did not alter any of our conclusions. If you are interested we have saved the calculations for non-parametric statistics but do not find it relevant to include it in the manuscript.
We believe the manuscript has improved and hope you find it suitable for publication.

On behalf of all the authors,
Yours sincerely,
Nana Wiberg and Magnus Jutell

1. Please ensure that your manuscript meets PLOS ONE's style requirements, including those for file naming. The PLOS ONE style templates can be found at https://journals.plos.org/plosone/s/file?id=wjVg/PLOSOne_formatting_sample_main_body.pdf

https://journals.plos.org/plosone/s/file?id=ba62/PLOSOne_formatting_sample_title_authors_affiliations.pdf

Done, please find the corrections accordingly

2. Please provide additional details regarding participant consent. In the ethics statement in the Methods and online submission information, please ensure that you have specified what type you obtained (for instance, written or verbal, and if verbal, how it was documented and witnessed). If your study included minors, state whether you obtained consent from parents or guardians. If the need for consent was waived by the ethics committee, please include this information.

Now specified in the text, as the first section under Procedures

3. We note that the grant information you provided in the 'Funding Information' and 'Financial Disclosure' sections do not match.

When you resubmit, please ensure that you provide the correct grant numbers for the awards you received for your study in the 'Funding Information' section.

We will do, funding removed from acknowledgements.

4. Thank you for stating the following financial disclosure:

"No only funding was payment for the analysis"

At this time, please address the following queries:

a) Please clarify the sources of funding (financial or material support) for your study. List the grants or organizations that supported your study, including funding received from your institution.

b) State what role the funders took in the study. If the funders had no role in your study, please state: "The funders had no role in study design, data collection and analysis, decision to publish, or preparation of the manuscript."

c) If any authors received a salary from any of your funders, please state which authors and which funders.

d) If you did not receive any funding for this study, please state: "The authors received no specific funding for this work."

Please include your amended statements within your cover letter; we will change the online submission form on your behalf.

Please, we have changed to 'no funding'. The extra costs for the analysis was not regarded as a grant. We have changed in the cover letter and in the submission form

5. Thank you for stating the following in your Competing Interests section:

"NO"

Please complete your Competing Interests on the online submission form to state any Competing Interests. If you have no competing interests, please state ""The authors have declared that no competing interests exist."", as detailed online in our guide for authors at http://journals.plos.org/plosone/s/submit-now

This information should be included in your cover letter; we will change the online submission form on your behalf.

6. In your Data Availability statement, you have not specified where the minimal data set underlying the results described in your manuscript can be found. PLOS defines a study's minimal data set as the underlying data used to reach the conclusions drawn in the manuscript and any additional data required to

replicate the reported study findings in their entirety. All PLOS journals require that the minimal data set be made fully available. For more information about our data policy, please see http://journals.plos.org/plosone/s/data-availability.

Upon re-submitting your revised manuscript, please upload your study's minimal underlying data set as either Supporting Information files or to a stable, public repository and include the relevant URLs, DOIs, or accession numbers within your revised cover letter. For a list of acceptable repositories, please see http://journals.plos.org/plosone/s/data-availability#loc-recommended-repositories. Any potentially identifying patient information must be fully anonymized.

Important: If there are ethical or legal restrictions to sharing your data publicly, please explain these restrictions in detail. Please see our guidelines for more information on what we consider unacceptable restrictions to publicly sharing data: http://journals.plos.org/plosone/s/data-availability#loc-unacceptable-data-access-restrictions. Note that it is not acceptable for the authors to be the sole named individuals responsible for ensuring data access.

We will update your Data Availability statement to reflect the information you provide in your cover letter.

[Note: HTML markup is below. Please do not edit.]

Reviewers' comments:

Reviewer's Responses to Questions

Reviewer #1: Jutell et al. have assessed the associations between maternal and cord blood 25(OH)D. Although this is interesting in an Arab population, the findings are not novel. The authors would benefit from performing a more extansive literature review to see that such an association has been reported many times previously, and referring to some of this literature.

Abstract

I would not typically use the word "excellent" to describe a correlation. Please reword in the title and elsewhere. Please use . for decimal place marker rather than 25(OH)D typically stands for 25-hydroxyvitamin D, not "vitamin D". Please correct in abstract and introduction. Please ensure all abbreviations are defined within abstract.

Corrected as suggested

The aims of the study are not entirely clear from the abstract - the background in the abstract discusses the unknown role of the placenta but this establishing this does not seem to be the aim of the study. Please provide a clear aim and the background should be relevant to this.

Thank you, we agree and have now removed the sentence 'transport over the placenta'. The aim of the study is stated in line 23-24 as previously.

"Of note, delivery by acute cesarean section in the second stage affected the correlation (r=0.644, p=0.024)". What is meant by "affected"? This is not scientific terminology.

Sentence reformulated

" Of the 303 women, 138 (47.7%) had vitamin D deficiency (defined as 25(OH)D<50nmol/L), whereas only 34 (13.8%) of UCB samples fulfilled the criteria for neonatal deficiency (25(OH)D<30nmol/L)." There were only 237 women with maternal and UCB samples so it is impossible to compare the two percentages of deficiency as it may not reflect the same set of women. Also why was a different cut-off used for maternal and fetal defiency?

We have calculated and described the new values for the group with paired samples. The definition of maternal and neonatal vit D deficiency is taken from the latest review we could find

Overall the results in the abstract would benefit from some consideration of what is relevant to present here as at the moment it feels like presentation of a random selection of results. This may reflect the lack of clear aims/objectives in the abstract however.

We have re-written

There is no conclusion to the abstract.

We think the abstract is clear after the above mentioned corrections and amendment of a conclusion.

Background

" During pregnancy, the vitamin is also thought to facilitate the transport of several nutrients across the placenta, including calcium (4)" Please list exactly which nutrients vitamin D facilitates the transport of across the placenta, and support with some primary evidence rather than a review article.

Thank you for making us aware. We now have decided to exclude this passage from the manuscript since it was vaguely formulated.

" Low vitamin D has been associated with increased risk for preeclampsia, small for gestational age fetuses, preterm rupture of membranes, and dystocia, but according to WHO, the evidence is low and more trials are warranted (5–7)." The authors reference here review and guidelines from 2007-2012. Please undertake a more up to date literature review with regards to this as there have been numerous observational studies and trial data reported in the last 10-15 years.

We have gone through the literature. The Cochran Report 2019 highlights the supplementation regime and the possible risk for the above mentioned complications. The results from Vivanti AJ et al. are the latest we could find - the references are now added but unfortunately, it really does not change our statement

Ref 8 is incompletely report.

We have now completed the reference, which after the update is ref 7.

"It is a general assumption that maternal levels primarily control fetal levels and that in women with vitamin D deficiency, the fetus will suffer from insufficient supply." I would strongly disagree that this is an assumption, there are many published studies showing a strong correlation between maternal and UCB 25(OH)D.

I would suggest review of 10.1210/clinem/dgac263 which shows maternal and offspring genetic variation in the vitamin D metabolism pathway affects UCB 25(OH)D.

We have changed the text and added some new references to your suggestion. It was a very nice comment and helped us figure out this vitamin's complexity and metabolism. Though far from being as advanced as the epigenetic studies, we still found our results important and essential for daily life in the clinic. And to our knowledge, only a few studies have been published investigating the correlation of maternal 25(OH)D and UCB 25(OH)D within this small timeframe. Also, as mentioned in the publication by Saraf et al. 2016 (ref 23 in the manuscript), they found that only 3 out of the 40 articles included in their systematic review, published from 1974-2012, concluded maternal 25(OH)D to be higher in the newborn compared to maternal levels.

"The definition of hypovitaminosis in pregnancy is 25(OH)D below 50nmol/l and in neonates below 30nmol/L." Please provide a reference for this.

Please also note that many guidelines and societies suggest different values to this for defining vitamin D deficiency. Please review.

Done, references added

The background does not give clear justification for the aims of the study. Also the primary aim of establishing the correlation between maternal and UCB 25(OH)D has been reported previously on many occasions. What is novel about the aims of this work?

The novelty of this study is: the number of patients, the short time frame upon measurement of maternal 25(OH)D and that we have investigated the correlation across all maternal levels. Another novelty is the investigation of factors associated with low vitamin and how they affect the maternal-UCB-correlation.

Methods

What is EMR?

Electronic medical record, changed to full word for better understanding.

Was is a interviewer directed questionnaire or a written questionnaire completed by the participant?

The mothers that were analphabetic were read the questionnaire aloud and gave oral answers that were recorded by staff. Mothers not speaking English, Urdu or Arabic were excluded from the study.

obstetrical is not a word

Thank you for making us aware, corrected

Was all data normally distributed? If not, what statistical methods was used?

With this number of patients, we regarded the data to be normally distributed. We did calculate with non-parametric statistics to show if there was a difference in the results we are presenting. The non-parametric statistics did not change our results and, thereby, our conclusions. This is also clear from Table 1. If you prefer, we can change the tables according to your question.

From where was maternal anthropometric/BMI data obtained
How was GDM/PET/PIH diagnosed?

Thank you for your questions. The BMI was as diabetes, hypertension in and/or PET documented in the electronic medical record. The BMI was recorded at every visit, and we decided to use the first recording. Any pre-existing diagnosis was also documented. All women in the Emirates are offered a 2 hour OGTT and if pathological fasting, one or two hour glucose values she is diagnosed with GDM. The cut-off values used in the hospital are replicated from RCOG, recognised world-wide. At every admission or visit the blood pressure was measured and recorded.

Results

Were those without complete maternal/UCB 25OHD similar to those with that data?

Yes, small differences only. Our aim with the first table is to show the demographics for all included women to demonstrate how it differs between the three groups of maternal vitamin levels. Not many studies have shown that before. In Table 2 we present totally different values i.e. the correlations where data are from the complete maternal/UCB cohort. For your insight we have attached a table with some of the demographic data calculated only from the complete maternal/UCB cohort.

Statistics

Vitamin D - Mother 3 groups			Vit,D,labour	Neonatal weight at labor (g)	Neonatal Vit D,venous
0-25	N	Valid	53	53	53
		Missing	0	0	0
	Mean		17,3909	3076,9811	28,1449
	Median		18,9600	3095,0000	27,9500
	Std. Deviation		4,79989	607,86143	9,61633
	Percentiles	25	14,2800	2755,0000	20,7750
		50	18,9600	3095,0000	27,9500
		75	21,0200	3500,0000	33,9550
25-49	N	Valid	58	58	58
		Missing	0	0	0
	Mean		36,4864	3195,9483	49,3833
	Median		35,4700	3210,0000	49,1600
	Std. Deviation		7,68052	518,96444	12,15186
	Percentiles	25	29,9075	2877,5000	40,4525
		50	35,4700	3210,0000	49,1600
		75	43,9500	3495,0000	56,0275
50+	N	Valid	126	126	126
		Missing	0	0	0
	Mean		82,6526	3109,2698	98,1053
	Median		79,7450	3140,0000	95,2600
	Std. Deviation		23,47383	459,34375	26,32236
	Percentiles	25	66,3800	2872,5000	81,3950
		50	79,7450	3140,0000	95,2600
		75	93,6700	3380,0000	112,0250

Why are all data in table 1 presented as mean (SD) and median (IQR). If normally distributed present as mean (SD), if not, present only as median (IQR).

Same answer as above: With this number of patients, the normality is not important for the tests. We did calculations with non-parametric statistics to show if there was a difference in the results we were presenting. The non-parametric statistics did not change our results and, thereby, our

conclusions. This is also clear from Table 1. If you prefer, we can change the tables according to your question.

What are the units for cord Ca2+

mmol/L. We have added the unit in the text.

Why is BMI presented stratified rather than as mean (SD) /median (IQR)?

We have recalculated BMI as a continuous variable, Table 1. For Table 2, we found it necessary and more understandable to keep the data as they are.

The data shown in table 1 suggested BMI did differ by 25(OH)D status, contrary to the statement in the text.

We disagree, Table 1 is to show the difference between the different groups of maternal levels, and Table 2 is to show the correlation between maternal and UCB 25(OH)D (these calculations are done only on the paired samples)

I would suggest rather than looking at % GDM/Caesarean by 25(OH)D grouping, that you examine 25(OH)D as a continuous variable by GDM y/n, LSCS y/n, PET y/n etc

Thank you for your input. GDM and CS are categorical variables, so we prefer to present data as already done and with percentages.

What does AS<7 mean?

Apgar score over 7 at 5 minutes. We have changed the text and tables for better understanding.

"Subdivision into the different levels of maternal 25(OH)D showed a consistent correlation in all three groups without a poorer correlation with low maternal values, Fig 1." This sentence does not make sense. It cannot be consistent and poorer. Please provide the r values.

Thank you for making us aware. We have made changes in the manuscript accordingly.

"Also, we could not show a correlation between UCB142 Ca2+ and vitamin D supplementation dose (n = 141)" - I would not expect this to be a linear correlation. Was neonatla hypocalcaemia more common in infants of mothers who did not use any supplementation?

No, and there was no neonate with hypocalcemia. Text with means and SD are now added to the result section.

We hypothesized that a high dose of maternal vitamin D supplementation could affect the UCB Ca2+. After looking through the literature we could find that recent meta-analysis and RCT:s could not find a correlation between dosage and neonatal Ca2+, supporting our results.

Discussion

I disagree with the comment that the correlation between maternal and UCB 25(OH)D is rarely described. I would suggest the authors undertake a further literature review on this. Maghbooli BMC Pregnancy 2007, Grant Pediatrics 2013, Song Public Health Nutr 2013 for starters.

Thank you for recommending these articles. We have now changed the text in the discussion to emphasize that few studies have investigated the correlation within our limited time frame and how maternal comorbidities and mode of delivery could affect the correlation.

Maghbooli et al.'s findings (in accordance with ours) found a significant correlation between maternal and UCB 25(OH)D. Still, they did not collect data regarding maternal comorbidities (GDM, HT/preeclampsia) and how it may have altered the correlation.

Grant et al. investigated UCB and newborn blood response to maternal vitamin D supplementation during pregnancy. As mentioned above, they did not calculate or collect data regarding maternal comorbidities and how it affected the correlation.

Song et al. investigated the correlation between maternal and UCB 25(OH)D and maternal 25(OH)D and neonatal anthropometric data.

" With maternal levels <50nmol/L no newborn was born with levels under 40.5nmol/L." This sentence does not agree with what is shown in your figure.

This is a very important point, and we feel very grateful to get the opportunity to correct the data. We have corrected it in the result section.

" The evidence for an association between low 25(OH)D in the mother and complications during pregnancy such as obesity, diabetes, preeclampsia and dystocia are for discussion accordingly to the latest statement from WHO." This sentence does not make sense, but it is also important to realise that the statement the authors refer to is over 10 years old and the body of literature on this topic has advanced substantially since that time.

We have corrected this, please find our comment to the suggestion under the background section

It is not clear to me what the relevance of a correlation between maternal and UCB 25(OH)D is stratified by these outcomes. What is more relevant is how the clinical outcome is related to maternal 25(OH)D as this would potentially offer a therapeutic intervention to prevent the outcome and/or reason to stress supplementation in that group to prevent neonatal vitamin D deficiency and associated hypocalcaemia.

We are thankful for your input. We hypothesized that although of a given maternal 25(OH)D level, the correlation to the UCB level could be influenced by maternal comorbidity, i.e., certain groups need different supplementation regimes. In Table 1, we show that the neonatal outcomes are the same across the groups of maternal vitamin D levels. Our results show that the correlation was enormously significant throughout maternal levels and comorbidities without differences. We find that this has clinical relevance and that our data and findings hopefully will arouse curiosity to further research the intricate relationship between maternal and fetal/neonatal 25(OH)D metabolism in subgroups with different comorbidities, not only based on the factual maternal level.

Further thought should be given to the weaknesses of the study. why did 25% not have paired samples for example? Did those included represent the local population?

As mentioned in the manuscript 66 samples were lost due to hemolysis of umbilical cord blood.

Of note, the order of authors on the entered data differs from that on the manuscript.

Thank you for making us aware. We have now made changes accordingly.

Reviewer #2

I am unclear about the originality of the study. These data have been reproduced previously in similar regions.

We do not agree. In the study by Izzeldin et.al (conducted in the same region as our study) there was no consistency in the time of measurement (from gestational week 1-37). We have a time frame of max 24 hours between maternal and UCB measurements. Also we were not able to find studies including maternal comorbidities when calculating the correlation between maternal and neonatal 25(OH)D values.

Despite this UVB variations, data on supplementation and robustness of assay measurement are major flaws of the study. A detailed questionnaire of maternal skintype, nutritional intake of vitamin D ,sartorial habits, sunscreen use and many othe factors are not included in the analysis

We are thankful for the comment but for this study it was not the aim to investigate the mentioned details. For the robustness of "assay measurement" we have now stated the specifics of Liaison 25-hydroxy-vitamin D TOTAL in the method section.

Methods of sampling are not described in detail.

We have changed the text for better clarification.

Reviewer #3:

The authors compare total vitamin D levels in 237 blood samples out of 303 women/newborn in a cohort with high frequency of deficiency. They find, as others, a correlation between mother and child levels with slightly lower levels in women.

I have some issues that needs to be addressed before publication

Title:
Superlatives in the title is unwarranted. I would suggest "Correlation between maternal and…..".

The title is corrected accordingly

"Fetal" is usually an unborn offspring that develops and grows inside the uterus, as umbilical cord is investigated, I would suggest the use the term "newborn".

We have had many discussions on this topic. Looking through the literature we find no consensus. Since the umbilical cord blood is blood with a closed loop circulation (placenta-fetus) we find it appropriate to mainly use umbilical blood and if needed use the term 'neonate'.

The cohort:
It is stated that all women coming for delivery were invited to participate, but how many declined or were excluded (i.e. is the cohort representative?).

We have clarified and mentioned the numbers in the results section.

Instead of stratificating the women in nationality I would have preferred accord to skin color and/or tradition with covered clothing.

It is a comment for discussion, and of course, we could have chosen to divide the women from the skin phenotypes. But what defines the types and the colors and underlying colors? If this should be done scientifically correctly, you have to use a dermatological test and definition, and you can not just use fair/dark, as in the study by Shahida Rabbani et al. 2019. They do not define fair/dark, so what is dark? We found it more scientifically correct to use nationalities, and as you see from Table 1, there is no

difference across the groups with different maternal 25(OH)D levels. The population in Ajman consists mainly of locals, people from Pakistan and the Philippines (as seen from the table). The first two populations wear covering close (Abaya) due to the same religious belief. Choosing nationality would give us an idea of the importance of potential underlying genetic factors (uptake and metabolization of the vitamin) and if food traditions had an impact on the correlation between maternal and UCB 25(OH)D levels. We have added text under discussion regarding this stratification.

To me it is a bit unclear when the maternal blood sample was performed. In the abstract it is stated “at the time of admission”, however no further details are provided. Are these hours, days before delivery?

Most of our patients were admitted during either the early stages of-, or active labor and the 25(OH)D was measured hours or even minutes before delivery. A few patients had complicated deliveries and the time of admission measurement to delivery was then of course prolonged. To our knowledge no participant had more than 24 hours between maternal and UCB measurements and since serum 25(OH)D variates very little during that time frame we did not find it relevant to measure maternal-to-UCB measurement time.

General:
A bit more discussion regarding previous findings regard vitamin D transfer through placenta is warranted. (Haddad JG Jr, Boisseau V, Avioli LV. Placental transfer of vitamin D3 and 25-hydroxycholecalciferol in the rat. J Lab Clin Med. 1971 Jun;77(6):908-15; Gupta, M. M., Kuppuswamy, G., & Subramanian, A. R. (1982). Transplacental transfer of 25-hydroxy-cholecalciferol. Postgraduate medical journal, 58(681), 408–410; Kiely M, O'Donovan SM, Kenny LC, Hourihane JO, Irvine AD, Murray DM. Vitamin D metabolite concentrations in umbilical cord blood serum and associations with clinical characteristics in a large prospective mother-infant cohort in Ireland. J Steroid Biochem Mol Biol. 2017 Mar;167:162-168 and especially all the work by Dr Jane Cleal from the University of Southampton).

Thank you for your suggestions. Unfortunately we could not find an abstract or full article for the article by Haddad et.al.

The articles from the other research groups have been added to the discussion section and more discussion regarding genetic and racial factors have been added.

Furthermore, I need some more stringency in the nomenclature between vitamin D2 (ergocalciferol) and vitamin D3 (cholecalciferol). In this context, were the supplementation I D2 or D3 form?

The supplementation given to the women who had previous check-ups in our antenatal clinic were prescribed cholecalciferol but the women who were

unbooked or booked at other clinics only self-reported whether they did/didnt take vitamin D supplementation without specifying D3 or D2. We have added text in the manuscript to further specify between cholecalciferol and ergocalciferol and total 25(OH)D. Thank you for your input.
