# Supplementary material for: Correlation between maternal and umbilical cord 25-hydroxy-vitamin D levels over a range of values. A prospective observational study from the United Arab Emirates
Source: PLoS One. 2024 Apr 23;19(4):e0299862. doi: 10.1371/journal.pone.0299862 (PMC11037530; doi:10.1371/journal.pone.0299862)
Supplement: Attachment — Submitted filename: Response to reviewers.docx [file pone.0299862.s002.docx]

Malmö 06-12-23

Firstly, we express our gratitude for your invaluable input and remarks. We have diligently tried to incorporate your feedback, implementing adjustments in the manuscript. As indicated, kindly review the latest modifications highlighted in red and strikethrough. We trust that these alterations have enhanced the manuscript, rendering it suitable for publication, and we appreciate your continued consideration.

Best regards

Magnus Jutell and co-authors

Reviewer #1: Thank you for valuable remarks.

Thank you for submitting the revised manuscript. I remained concerned by the originality of this work, and do feel there is some "clutching at straws" to utilise some data. I remain uncertain as to how looking at the correlations stratified by maternal demographics is actually useful unless you are perhaps suggesting that this may influence the pathway of maternal-fetal 25D transfer. If this is the case, then some discussion of this and hypothesis around how the factors would influence this would actually make the manuscript far clearer. Furthermore, to be able to state there was no difference between the correlation, a statistical correlation needs to be made between them. I would suggest discussions with a statistician as to how to achieve this.

-       Compared to the published advanced epigenetic studies, we are aware of the simplicity of our research. However, it does not imply that our results are ignorable and uninteresting. In our experience, we are moving from generalization to personalized medicine. The placenta plays an essential and active role in the transition and activation of 25(OH)D. Still, it is not known how or whether the 25(OH)D pathway is altered with certain placenta pathologies seen in, for example, diabetes, obesity, and preeclampsia. We found one publication that studied the role of an abnormal placenta and the expression of vitamin D receptors, which led us to this study. As for today, the women are all treated the same without considering if some of the most common maternal comorbidities affect the pathway, i.e., maybe some subgroups have special needs to achieve the optimal level of maternal and fetal 25(OH)D. This study shows that maternal and fetal 25(O)D strongly correlates with all vitamin D levels (which has not been shown before) and that the correlation is consistent through common maternal comorbidities, implying that if we know the mother's status, we can estimate which baby is at high risk. In epidemic areas for low 25(OH)D, where the resources are limited, we need to know which mothers and babies are at exceptionally high risk of 25(OH)D deficiency to optimize screening and treatment. Our results assume some upregulating of the placenta function in mothers with comorbidities, either due to increased VDRs or to a more complex epigenetic alteration. We have added text about this in the introduction and discussion. Regarding the comment about statistics, we have calculated new statistics with our co-author (an experienced statistician) and added them to the text and Table 2.

* As per my previous comments, please rephrase the term "excellent correlation" in the abstract and elsewhere in the paper.

*-	Thank you for making us aware. Changes have been made accordingly.*

* Except for acute cesarean section in the second stage (p=0.037), the correlation remained strong after dividing the women into subgroups of supplementation intake, BMI, diabetes, hypertension/preeclampsia, and time spent outside (p<0.001)." I do not understand this sentence and what the authors are trying to show/what analysis has been done or why.

*-	We have tried to present different factors that potentially could affect the pathway/correlation between maternal and UCB 25(OH)D. After further discussion with our statistician (and co-author as mentioned), we have now made calculations to compare the significance between the different correlations to better compare the subgroups with each other. Please see the new text in the result section line 220-230 and in Table 2.*

* Overall I find the abstract very difficult to follow. It seems that the authors have run a large number of analyses of different things - maternal/UCB correlations, possibly then across different subgroups, maternal 25D with different outcomes, and then chosen the significant factors to report. Please rewrite the abstract giving clear and exact aims, and address these in order within the abstract. eg "Of those with paired samples, 111 (46.8%) women had vitamin D deficiency (defined as25(OH)D<50nmol/L), whereas only 32 (13.5%) of UCB samples fulfilled the criteria forneonatal deficiency (25(OH)D<30nmol/L). Low maternal 25(OH)D was associated with significantly more women having hypertension/preeclampsia." Why is this all presented when the abstract states the aim is "This prospective observational study aimed to correlate the maternal 25(OH)D levels to UCB levels in ahigh-risk population and to show if certain factors influenced the correlation

-	We have changed the abstract. We need to mention that we have not chosen only significant data from our results, but we have chosen from aims and subjects. The Tables were drawn before the study began. We have a lot of data not presented in this article (for example, the odds for different diagnoses with vitamin D status) for better qualification and stratification aligned with our aims.

"Introduction"Accordingly to a study from 2012 there is only limited amount of data when
52 it comes to the evidence for the levels and the correlations between maternal and neonatal
53 25(OH)D" I dont understand the first part of this sentence. Levels of what?

-	*We have now changed the introduction to better describe our aims. Please see changes.*

* It would be helpful to add some comment as to why you think any of these maternal factors would influence the correlation, and what the value in knowing this information would be.

*-	Thank you for your guiding comments. We agree! We have now added our hypothesis on why we believe these factors potentially could be of benefit and value. Please see the changes under introduction.*

Results

* Please add units of measurement to table 1

*-	Thank you for making us aware.*

* Please review the numbers in () for HT/PET in table 1 - i do not think these are correct, and assuming these represent the % of women, do not add up to 100

*-	Thank you for making us aware! Please see changes in red.*

* "In contrast there was a trend towards more women with preeclampsia/hypertension with lower 25(OH)D levels," It is not a trend towards, there were more women with PET/HT in those with lower 25D

*-	A good point! Changes have been made accordingly.*

* "The correlation was strong, and with no differences in correlation coefficients for the different subgroups of maternal 25(OH)D levels." Have you performed a statistical test to be able to categorically state there is no difference between these correlations? I would recommend discussion with a statistician. This should be done for all the subgroup analyses that have been undertaken. I do not think the conclusions that have been made that these subgroups did not affect the correlation can be made until this has been performed.

- *Thank you for your remark. We have now conducted “test of difference between correlations using Fisher's z-transformation with Bonferroni correction of p-values” to better compare the correlations with each other. Please see the changes under results.*

* "The scientists behind the meta-analysis..." the findings dicussed are not from the meta-analysis but from a different paper, as referenced.

-	*We have now sorted this. Thank you for your input.*

* I disagree that normality can be assumed based on the n number. This is not always the case, and even independent of n, some data will not be normally distributed. The descriptive data should be presented as either mean (SD) if normal on visual inspection and median (IQR) if not normal on visual inspection.

- *You are quite correct that n does not necessarily predict if the data is normally distributed or not. However, one-way ANOVA works perfectly fine on this data set regarding that even if the original data is not normally distributed the mean values will be. In table one we have presented all the measurements with both IQR and SD and we still find it suiting to do so.*

Reviewer #2: I do not fell that this study has high standards for publication since similar data have been previously reported

- *We valued your input during the first review and we are sorry that you feel this way though we do not agree. Some studies show a correlation but whether this correlation in influenced by comorbidities or demographics (except sunlight) have not been published to our knowledge and therefore we would be grateful if you can share those publications with us. Please let us know if you have changed your mind after reading the second draft of the manuscript.*

Reviewer #3: Most of the issues has been clarified, however, some still needs to be addressed.

Generally, the manuscript could benefit from a native English speaking review, I can recommend American Journal Editors (AJE). Alternative, a careful proof reading would make the manuscript more readable.

- *We have involved a native speaker to bring better clarity in our text. Hope you find the changes suitable.*

Title: Abbreviation 25(OH)D is not feasible in the title (unexplained).

-	*Thank you for making us aware! Changes have been made accordingly.*

Affiliation: Usually department, institution, city, country is provided, this manuscript lacks some city and one report zip code. Uniformity would be prudent.

-	*Thank you for pointing that out. The first author’s affiliation has changed after finishing medical school. Also, there are no zip code available for the hospital in Ajman.*

Short title: The nomenclature regarding fetal, newborn, neonatal is still not uniform (ex: in the short title “fetal” is used), as well as other places I the text.

-	*Thank you for the feedback! We have now chosen to use “umbilical cord blood (UCB)” when describing correlations of 25(OH)D and the term “neonatal” when describing data and characteristics from the baby, thus excluding the term newborn entirely. We hope that this accommodates your concerns. Please see the changes in the text.*

Abstract:
Abbreviations in the abstract should be explained at firsts occurrence (25(OH)D), it is first explained at line 54.

-	*Please see changes in the abstract.*

Is p-values below zero? (p<0,000), write p<0.001 (use punctuation instead of comma). Also, present in table 2.

- *Thank you for noticing! We have made changes accordingly.*

What is “paired samples”?, is not all comparison on paired (mother/child) samples, or is groups compared?

- *Thank you for making us aware. We have added an explanation for this on the method section under “procedure”*

Material and Methods:

The abbreviation UAE should be explained, but as it is not used frequently, just write United Arab Emirates.

-	*A good point! Changes have been made.*

Correct “after vaginal and cesarean section” to “after vaginal or cesarean section”.

-	*Done.*

I presume that the vitamin D analyses were performed on a Liaison XL instrument with the LIAISON® 25 OH Vitamin D TOTAL Assay?

-	*You are quite correct! We have made the suggested changes.*

Table:
IQR is the interquartile range (Q3-Q1), a single cipher. However, two ciphers are presented, I assume it is Q1 and Q3. Correct IQR to first and third quartile or correct the ciphers.

-	*We have now made changes in table 1 and presents IQR as a single cipher.*

Figure 1&2:
Please modify the designation of the axis, fetal/neonate, vitD labour, at least make them uniform.

-	*Thank you for letting us know. Changes have been made.*

SPSS is cited correctly as: IBM SPSS Statistics for Windows, Version 28.0. Armonk, NY: IBM Corp

-	*Thank you for letting us know. Changes have been made according to your suggestion.*
