# Supplementary material for: Correlation between maternal and umbilical cord 25-hydroxy-vitamin D levels over a range of values. A prospective observational study from the United Arab Emirates
Source: PLoS One. 2024 Apr 23;19(4):e0299862. doi: 10.1371/journal.pone.0299862 (PMC11037530; doi:10.1371/journal.pone.0299862)
Supplement: Attachment — Submitted filename: Last Answer 3 to reviewers _ Nw.docx [file pone.0299862.s003.docx]

Reviewer #1: The manuscript is much improved and more clear to follow. There are some minor points below regarding the main text but the abstract does require further modification.

Abstract:
*-" This prospective observational study aimed to study the correlation between maternal and UCB 25(OH)D levels in a population with high risk for 25(OH)D deficiency, secondary, to show if maternal demographics or comorbidities certain factors influenced the correlation." Please review this sentence and ensure it makes sense. I am unclear why the word secondary is included.

*- Similarly, this sentence does not make sense in English "At admission for labor, respectively,
after delivery, 25(OH)D was analyzed in maternal serum and in UCB"

*- I remain unclear as to what "analyzing for different maternal demographics means" - do you mean you undertook stratified analysis by these characteristics or you looked for a statistical interaction?

*- Line 43 - a p value of 0.037 is significnat. Please explain why this was not significant?

*- Line 44-45 - what are the actual r values for these correlations? Just giving a p value is meaningless.

**** Thank you for the comments and the careful review. We have changed the abstract as suggested, and we hope you agree on an improvement/clarification. We have not written the specific r or p values for each analyzed parameter since we found it was too many values to outline. Therefore, we chose only to mention the lowest value. *We changed strong to very strong accordingly to the recommendations from BMJ* *(If we wish to label the strength of the association, for absolute values of r, 0-0.19 is regarded as very weak, 0.2-0.39 as weak, 0.40-0.59 as moderate, 0.6-0.79 as strong and 0.8-1 as very strong correlation, but these are rather arbitrary limits, and the context of the results should be considered).*

-Methods Line 135 - the use of respectively in this sentence is incorrect. This is true in other places in the manuscript also.

*Thank you for pointing that out. We have made changes under the methods section and in the rest of the text.*

-Results Line 162 - should be "were diagnosed"

*Done! Thank you.*

- The text in paragraph lines 167 needs to refer to the table

*Agree. We have made the changes accordingly.*

- Why is the data in Table 1 presented as both mean(SD ) and median (IQR) - please prevent the relevant statistics depending on the distribution of the data. Similarly please refer to the figures in the relevant text.

*As per our previous answer to this question we have presented all the measurements with both IQR and SD in table 1 and we still find it suiting to do so. One way ANOVA is suitable for this kind of calculations regardless of normal distribution or not, because the mean values will be. After discussions with our statistician, we still feel confident in the way that the data is presented.*

- I am really not sure of the point of comparing the correlations of vitamin D supplementation to women with "unknown supplementation". Please justify as to me this seems meaningless given the potentially very mixed nature of this group.

*A good point! We have compared all the correlations in table 2 using Fisher´s z-transformation with Bonferroni corrections of p-values and choose to present the significant findings. We still feel that it is important to present the significant data to be stringent to our aims - but also, that it should be interpret with caution.*

*We are aware of the potentially mixed nature in the “unknown” group of 25(OH)D supplementation and the difficulty to draw any conclusions from our calculations in this group. We have therefor put this result into context, in the discussion and conclusion section, where we have concluded that it is not possible to draw any conclusions regarding this comparison.*

- line 220 - a p value of 0.037 is typically considered statistically signficiant although i agree we should not a slave to the p value. Authors have also stated "All tests were two-tailed, and a P-value < 0.05 was
152 considered statistically significant.". PLease review this and the discussion accordingly.

*You are quite correct. Thank you for pointing that out. The correlation coefficient became weaker in the second stage caesarean group compared to the other delivery modes, but the p-value is still significant (<0.05) and there were no significant differences between the correlations within this group. We have changed the manuscript accordingly.*

line 275 do you mean UCB 25(OH)D here - UCB needs to be collected immediately so it is unlikely to lead to heterogenous time interval between maternal and offspring blood sampling.

*We were referring to the maternal measurement of vitamin D. We have changed the sentence for better clarity. Thank you.*

-lines 334 - do you think that maternal haemodilution around the time of delivery could be contributing to the relatively lower 25OHD in the mother compared to infant. Additionally how many women received iv fluids during delivery which could have contributed to a dilutional effect?

That is an exciting thought. The analysis was done at admission and before any IV fluids were given, i.e., this is not the reason, and also, therefore, we found it means less for this study to look into IV fluids given during delivery. Even the cases with cesarean section are unaffected because the test was done before they finally ended up with CS. According to a small study from Japan (50 participants, [PLoS One.](https://www.ncbi.nlm.nih.gov/pmc/articles/PMC7531781/) 2020; 15(10): e0239954) there is a decrease in Hb, Hct during pregnancy, but another study (Clin Chem Lab Med 2010 Feb;48(2):237-48) found Hb and Hct to be stable during pregnancy and at partus.

Reviewer #3: The manuscript has improved, however, it still is hard to read (especially the abstract).
I would have used other phrases such as total instead of "cumulative vitamin D2 & D3".
Plasma, serum and blood are mentioned as source of measurement. Is that correct?
The interesting discussion is a bit speculative based upon the minor difference in mother/offspring vitamin D levels.

*Thank you for your feedback on the manuscript. We have incorporated your suggestions to improve the abstract. Regarding the nomenclature for the source of 25(OH)D measurement, there is variation in the literature, with some authors referencing plasma 25(OH)D and others serum 25(OH)D. While plasma contains clotting factors and serum does not, our understanding is that this difference does not impact total 25(OH)D levels. Following your thorough review, we have refined the terminology to exclusively reference serum and blood, when appropriate, in accordance with the design of LIAISON® 25 OH Vitamin D TOTAL, which measures total 25(OH)D in serum.*

*We are grateful for your interest in our discussion. We have diligently contextualized our results and proposed hypotheses to why it varied from previous studies. While we acknowledge some speculative aspects, we have exercised caution, refraining from drawing absolute conclusions given the nature of our prospective observational study and the size of our study population.*
